# Machine learning applications in studying mental health among immigrants and racial and ethnic minorities: an exploratory scoping review

**DOI:** 10.1186/s12911-024-02663-4

**Published:** 2024-10-10

**Authors:** Khushbu Khatri Park, Mohammad Saleem, Mohammed Ali Al-Garadi, Abdulaziz Ahmed

**Affiliations:** 1 Department of Health Services Administration, School of Health Professions, University of Alabama at Birmingham, 1716 9th Ave S, Birmingham, AL 35233 USA; 2 Department of Biomedical Informatics, School of Medicine, Vanderbilt University, 1161 21st Ave S # D3300, Nashville, TN 37232 USA; 3 Department of Biomedical Informatics and Data Science, Heersink School of Medicine, University of Alabama at Birmingham, Birmingham, Alabama 35233 USA

**Keywords:** Machine learning, Mental health, Minorities, disparities, review

## Abstract

**Background:**

The use of machine learning (ML) in mental health (MH) research is increasing, especially as new, more complex data types become available to analyze. By examining the published literature, this review aims to explore the current applications of ML in MH research, with a particular focus on its use in studying diverse and vulnerable populations, including immigrants, refugees, migrants, and racial and ethnic minorities.

**Methods:**

From October 2022 to March 2024, Google Scholar, EMBASE, and PubMed were queried. ML-related, MH-related, and population-of-focus search terms were strung together with Boolean operators. Backward reference searching was also conducted. Included peer-reviewed studies reported using a method or application of ML in an MH context and focused on the populations of interest. We did not have date cutoffs. Publications were excluded if they were narrative or did not exclusively focus on a minority population from the respective country. Data including study context, the focus of mental healthcare, sample, data type, type of ML algorithm used, and algorithm performance were extracted from each.

**Results:**

Ultimately, 13 peer-reviewed publications were included. All the articles were published within the last 6 years, and over half of them studied populations within the US. Most reviewed studies used supervised learning to explain or predict MH outcomes. Some publications used up to 16 models to determine the best predictive power. Almost half of the included publications did not discuss their cross-validation method.

**Conclusions:**

The included studies provide proof-of-concept for the potential use of ML algorithms to address MH concerns in these special populations, few as they may be. Our review finds that the clinical application of these models for classifying and predicting MH disorders is still under development.

**Supplementary Information:**

The online version contains supplementary material available at 10.1186/s12911-024-02663-4.

## Introduction

Common Mental Disorders (CMDs), including major depressive disorder, mood disorder, anxiety disorder, and alcohol use disorder, affect approximately one in five people worldwide [1, 2]. More specifically, the global prevalence of post-traumatic stress symptoms is 24.1%, anxiety is 26.9%, sleep problems are 27.6%, depression is 28.0%, stress is 36.5%, and psychological distress is 50.0% [3]. Post-COVID, the World Health Organization estimates that there has been further worsening of mental health status with a further 25% increase in depression and anxiety disorders [4].

Mental health (MH) disparities are significantly influenced by stigma, discrimination, and socioeconomic challenges [2, 5]. These disparities are exacerbated for minority populations who often face limited access to MH services due to geographic, economic, and literacy barriers, leading to lower satisfaction with healthcare and higher dropout rates from MH services compared to Whites [6–10]. Black and Latinx individuals, for example, are at higher risk of persistence and disability from CMDs [11–13]. While Asian Americans are considered to have better MH status compared to Whites and other racial and ethnic minorities, this is poorly studied [14]. Immigrants often experience a temporary improvement in MH upon arrival, known as the “immigration paradox”, but their MH deteriorates over time due to assimilation stresses, racism, discriminatory and exclusionary policies, status loss, and sometimes violence [5, 15–18]. Refugees face significantly higher rates of severe psychiatric disorders, including post-traumatic stress disorder, due to adverse pre-migration conditions [16, 19].

Ultimately, CMDs and other MH conditions may disproportionately affect ethnic and racial minorities overrepresented in homeless, incarcerated, and medically underserved populations [20], and thus there is a need to there is a need to understand and strengthen the MH resiliency of these populations. Clinicians and researchers have increasingly collected “big data” to aid this mission. This includes structured and unstructured data from electronic health records (EHR), smartphones, wearables, social media, and other large, complex sources. While traditional epidemiological methods have proven highly effective in analyzing complex data in MH research, machine learning (ML) approaches can offer complementary tools that can potentially enhance the ability to identify subtle patterns and relationships, particularly in these large, multidimensional datasets. A combined approach may reveal additional insights into MH disparities across various populations, leveraging the strengths of both traditional and ML-based analytical techniques.

Machine learning encompasses a variety of algorithms and statistical models that enable programs to improve their performance on a task through experience. In the context of MH research, ML techniques can be broadly categorized into supervised learning, where models are trained on labeled data to predict outcomes, and unsupervised learning, which identifies patterns in unlabeled data [21–24]. The application of ML in health sciences, including mental health, has been growing. ML models have been developed to predict risk scores for various mental health conditions, potentially aiding in diagnosis and screening [25–27]. While several reviews have discussed ML applications in mental health research [28, 29], there’s been limited focus on how these models address factors such as race, ethnicity, or immigration status. For example, Maslej et al. [30] conducted a rapid review using a Critical Race Theory perspective to examine how race and racialization are defined in ML applications for Major Depressive Disorder, but their study did not extend to other common mental disorders or broader mental health issues.

This study encompasses a broad spectrum of mental health conditions, ranging from CMDs to less prevalent but equally critical conditions such as schizophrenia, bipolar disorder, and personality disorders. We also consider related issues like suicidality and juvenile delinquency, which, while not psychiatric disorders themselves, are often associated with mental health challenges. This comprehensive approach allows us to explore how machine learning (ML) can support various aspects of mental health care across diverse conditions and populations. By expanding our focus beyond CMDs, we acknowledge the unique challenges in diagnosis and management presented by different mental health conditions, particularly in vulnerable populations such as immigrants, refugees, and minorities. This broader scope ensures a more inclusive examination of how ML can be applied to improve mental health care across the full range of diagnostic categories and related issues.

Our search terms reflect this comprehensive approach, including both specific psychiatric diagnoses and related mental health conditions. This allows us to capture the full potential of ML applications in mental health, from common disorders to more complex and less frequent conditions, providing a thorough exploration of the field’s current state and future directions.

This review asks: What is the breadth of existing literature on the application of ML techniques for addressing MH challenges in vulnerable populations of immigrants, refugees, migrants, and racial and ethnic minorities? This study also examines the feasibility of implementing ML solutions in MH, focusing on how ML integration affects the workload of healthcare professionals and analyzing improvements in patient care by ML. Our study aims to build upon existing research by examining ML applications across a wider range of mental health conditions, with a specific focus on how these models account for and perform across diverse populations, including racial, ethnic, and immigrant groups.

## Methods

Two reviewers (KP and AA) independently conducted searches in Google Scholar, EMBASE, PsycINFO, and PubMed from October 2022 to March 2024. All queries had three components: an ML-related term (e.g., “machine learning,” “artificial intelligence”), an MH-related term (e.g., “mental health,” depression, “post-traumatic stress disorder”), and a population of a focus search term (e.g., immigrant, refugee, minority*). These terms were combined with the Boolean operators to create final search strings. Queries were conducted on titles, keywords, and abstracts. Backward reference searching was also conducted, reviewing references from the articles that matched our search criteria for more articles that could fit our inclusion criteria. See the Appendix for full query syntax.

Inclusion criteria included: (i) the article reported using a method or application of ML in an MH context; (ii) the primary population studied was immigrants, refugees, migrants, and/or racial and ethnic minorities; (iii) the article was published in a peer-reviewed publication; (iv) the article was available in English. We did not limit articles to just those published in America. Due to the rapid advancements in ML, we limited our search criteria to articles published after 2014. Articles were excluded if they were narrative (e.g., commenting on future applications of ML in MH or were not empirical) or if they did not exclusively focus on a minority population from the respective country (e.g., a study of ethnically Chinese migrants in China would be excluded). Conflicts over inclusion were discussed, and a consensus was sought before the inclusion or exclusion of the publication in question.

Data were extracted from each article, including study context, the focus on mental healthcare, sample, data type, type of ML algorithm used, and algorithm performance. A narrative synthesis approach was applied.

## Results

To summarize the results of this review, we present them in three sections. The first section includes the results of the selection process. The second section details the characteristics of the selected studies, such as their area of focus, publication year and location, and data source. The third section highlights the machine learning models used in the studies for predicting and studying mental health outcomes.

### Selected studies

**Fig. 1:**
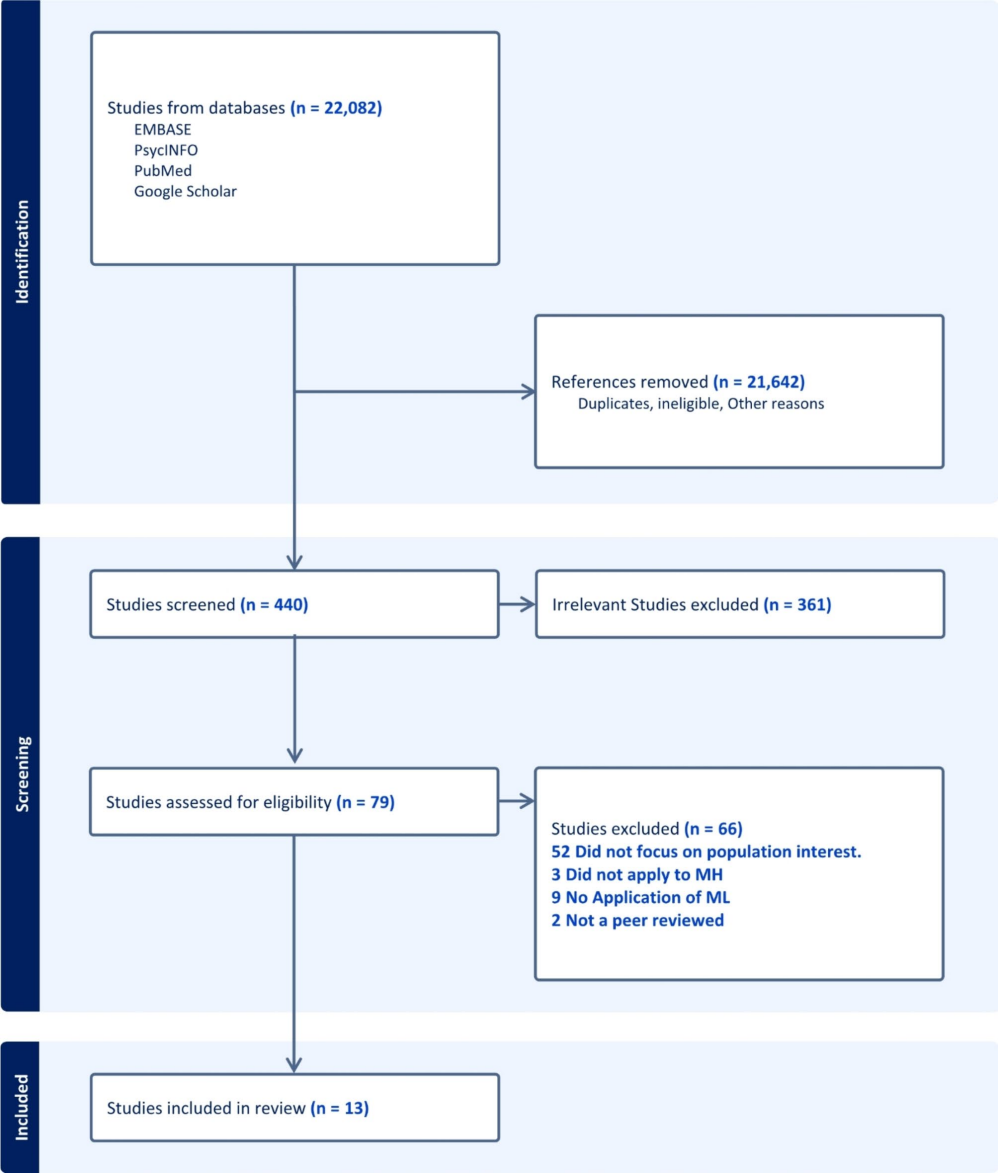
Study selection

Our search strategies resulted in a total of 22,082 listed articles from Google Scholar, EMBASE, PsycINFO, and PubMed. Figure 1 shows the flow of our search strategy and results. All records from PsycINFO and PubMed were reviewed, an additional 280 records were reviewed from Google Scholar, and the most relevant 100 were reviewed from EMBASE. Based on titles and abstracts, 79 were selected and further reviewed. Most of these records were excluded because they did not focus on the population of interest. Instead, they focused on majority populations and racially homogenous populations and/or did not include discussions about immigrant/migrant status. We also reviewed five abstracts from citation searching. Ultimately, 13 publications were included in this review.

In our scoping review, we also identified several gaps that have significant implications for the field of MH research using ML. There is a lack of data availability, especially longitudinal data, which is important for developing predictive models. Most of the studies focus on well-represented groups, leaving the minority population underrepresented, which can lead to biased algorithms and unjust health outcomes. These gaps underscore the need for targeted efforts to broaden the scope of research in this dynamically evolving field.

### Publication characteristics

Surveys [31–34], drawings [35], secondary data sets (including EHR data, surveillance data, and national sample sets) [35–39], internet-based posts [40, 41], and genomic sequencing data [42, 43] were analyzed in the included publications (see Table 1). Various populations were considered, and sample sizes varied widely due to the type of data collected and analyzed. For example, Augsburger and Elbert [31] enrolled 56 resettled refugees in a study to prospectively analyze their risk-taking. Goldstein, Bailey [37] used a retrospective dataset with 22,968 unique Hispanic patients, and Acion et al. [36] included 99,013 Hispanic individuals in their secondary data analysis. Children were also included in the reviewed studies; one examined the depression and PTSD levels of 631 refugee children residing in Turkey [34]. Another study analyzed drawings from 2480 Syrian refugee children to find the predictors of exposure to violence and mental well-being [35]. Other sample sets analyzed 0.15 million unique tweets from Twitter [40] and 441,000 unique conversations from internet message boards and social media sites [41]. Genomic sequencing data was collected from 4,179 Black individuals [43] and 524 Black individuals [42].

Most reviewed studies used supervised learning intending to explain or predict certain MH outcomes. For example, to classify substance use disorder treatment success in Hispanic patients, Acion et al. compared 16 different ML models to an ensemble method they called “Super Learning” [36]. Similarly, Huber et al. compared various ML algorithms, including decision trees, support vector machines, naïve Bayes, logistic regression, and K-nearest neighbor, to determine the model with the best predictive power for classifying schizophrenia spectrum disorders in migrants [38]. Two studies explored the impact of trauma exposure on MH using ML [31, 35]. Two studies utilized social media data to understand MH at a population-health level through ML algorithms [40, 41]. All study aims are found in Table 1.

**Table 1:** Publication characteristics of included studies

First Author (year)	Study Aim	Area of MH focus	Sample size and characteristics	Data analyzed
Acion (2017) [36]	Predict substance abuse treatment success using 17 different machine learning models	Substance abuse	99,013 Hispanic individuals	TEDS-D 2006–2011
Augsburger (2017) [31]	Assessed risk-taking behavior in refugees after exposure to trauma using a gamified BART	Trauma	56 Refugees resettled in Germany	Surveys and data on BART
Baird (2022) [35]	Used drawings by refugee children to estimate predictors of exposure to violence and mental wellbeing	Trauma	2480 Syrian refugee children	USF 2016 dataset
Castilla-Puentes (2021) [41]	To understand how Hispanic populations converse about depression by conducting big data analysis of digital conversations through machine learning	Depression	441,000 unique conversations about depression; 43,000 (9.8%) conversations were by Hispanics	Conversations from open sources like topical MH websites, message boards, social networks, and blogs
Choi (2020) [32]	Examined the predictive ability of discrimination-related variables, coping mechanisms, and sociodemographic factors on the psychological distress level of Korean immigrants in the U.S. during the pandemic	Psychological distress	790 Korean immigrants, foreign and US-born	Surveys
Drydakis (2021) [33]	Understanding associations between the number of mobile applications in use aiming to facilitate immigrants’ societal integration and increased level of integration, good overall health, and mental health	Depression	287 immigrants in Greece	Surveys
Erol (2022) [34]	Examine the PTSD and depression levels of Syrian refugee children and adolescents, the difficulties they experienced in access to food and education, and the changes in their family income, and evaluate the effects of these factors on symptom severities of depression and PTSD	Depression & PTSD	631 Refugee children living in Turkey	Surveys
Goldstein (2022) [37]	To examine the relationship between experiencing discrimination and suicidal ideation in Hispanic populations	Suicidal ideation	22,968 Hispanic individuals	Holmusk and MindLinc EHR datasets, 52,703 patient-year observations from 2010 to 2020
Haroz (2020) [39]	Develop a model using ML methods to better identify those at highest risk for suicide in Native American communities	Suicidal ideation	2,390 Native American individuals	Surveillance program data
Huber (2020) [38]	Differentiated native Europeans and migrants as to their risk of having schizophrenia	Schizophrenia	370 patients with diagnosed schizophrenia spectrum disorder	Hospital data from 1982 to 2016
Khatua (2021) [40]	Using social media data to identify the voices of migrants and refugees and analyze their MH concerns	General MH	0.15 million tweets, 2% from self-identified refugees	0.15 million tweets
Liu (2021) [43]	Used ML algorithms to distinguish ADHD, depression, anxiety, autism, intellectual disabilities, speech/language disorder, delays in development, and oppositional defiant disorder in Blacks using the data from their genome.	ADHD, depression, anxiety, autism, intellectual disabilities, speech/language disorder, delays in development, oppositional defiant disorder	4179 Black individuals	Genomic sequencing data
Liu (2021) [42]	Used ML algorithms to distinguish ADHD in Blacks using the data from their genome.	ADHD	524 Black individuals	Genomic sequencing data

Table 2 presents some high-level characteristics of the reviewed publications. All but two of the analyzed articles were published in the last three years, with the two earliest from 2017 [31, 36]. More than half of the papers were from the US or incorporated populations based in the US, four were from Europe, and the rest were from Asia. Among the 13 articles, five focused on refugee populations [31, 33–35, 40], three focused on Hispanic populations in the US [36, 37, 41], two focused on Black individuals [42, 43], one on Native Americans [39], and the last two articles focused on Korean immigrants in the US [32] and immigrant populations in Europe [38]. The areas of mental health focus included stress [40], ADHD [42, 43], trauma [31, 35], depression [33, 41, 43], PTSD [34], psychological distress [32], schizophrenia [38], suicidal ideation [37, 39], and substance abuse [36].

**Table 2:** Publication analysis

Characteristic	*N*	%	Reference
**Year of publication**	*N* = 13		
2017	2	15.4%	[31, 36]
2020	3	23.1%	[32] [38] [39]
2021	5	38.5%	[33, 40–43]
2022	3	23.1%	[34, 35, 37]
**Region**	*N* = 13		
Asia			
Turkey	1	8.3%	[34]
Jordan	1	8.3%	[35]
Europe			
United Kingdom	1	7.7%	[33]
Germany	2	15.4%	[31, 40]
Switzerland	1	7.7%	[38]
USA	7	53.8%	[32, 36, 37, 39, 41–43]
**Population of focus**	*N* = 13		
Refugees	5	38.5%	[31, 33–35, 40]
Hispanics	3	23.1%	[36, 37, 41]
Native Americans	1	7.7%	[39]
African Americans	2	15.4%	[42, 43]
Korean immigrants	1	7.7%	[32]
European immigrants	1	7.7%	[38]

### Machine learning model performance and characteristics

Table 3 outlines a summary of ML characteristics and model performance. This review found that all 13 included publications fell into three categories: classification [32, 36–40, 42, 43], regression [31, 33–35], and unsupervised topic modeling [41].

**Table 3:** Machine learning model characteristics from selected articles

First Author (year)	Outcome Variable	Predictors (Input variables)	ML technique	Cross-validation method (internal, external)	Type	Program used	Best algorithm performance
Acion (2017) [36]	Substance abuse treatment success	28; 10 patient characteristics, 3 treatment factors, referral type, problematic substance characteristics, and mental health problem	LR, RLR, Lasso-LR, EN, RF, DNN, EL	Two-fold cross-validation (I)	Classification	R; H2O R interface and package rROC	AUC: 0.793–0.820 Best mode: EL
Augsburger (2017) [31]	Risk-taking behavior as measured using a balloon analog risk task (BART)	Exposure to different types of childhood maltreatment, experiences of war and torture, lifetime traumatic events and symptoms of depression and PTSD, sociodemographic factors	Stochastic GBM	Tenfold cross-validation with three repetitions (I)	Regression	R; *gbm* & *caret*	RMSE: 18.70, R^2: 0.20,
Baird (2022) [35]	Psychological trauma as measured on the GHQ-12	18 digitally coded features in self-portraits and free drawings	One model method used: LASSO-R	K-fold cross-validation (I)	Regression	Not reported	R-squared: 0.108
Castilla-Puentes (2021) [41]	Tone, topics, and attitude of digital conversations	Digital conversations	NLP and texting mining	Not used	Unsupervised- Topic modeling	CulturIntel	Not reported
Choi (2020) [32]	Psychological distress is measured using the Kessler Psychological Distress Scale (K10)	Demographic characteristics, three types of discrimination characteristics, three types of coping mechanisms	ANN	Not used	Classification	SPSS	AUC: 0.806
Drydakis (2021) [33]	Increased level of integration, overall health, and mental health	Number of mobile applications in use that facilitate immigrants’ societal integration	Linear Regression	Not used	Regression	Not reported	*p* < 0.005
Erol (2022) [34]	Symptom severity of depression and PTSD	Demographic data, PTSD and depression levels, access to food and education, and changes in family income	Linear regression	Not used	Regression	SPSS	R-squared = 0.123
Goldstein (2022) [37]	Suicidal ideation in the past year	Experience of discrimination, demographics	Deep-learning NLP algorithms and LR	Not used	Classification	Not reported	Not reported
Haroz (2020) [39]	Suicide attempts, measured at 6, 12, and 24 months after an initial suicide-related event	73; demographic characteristics, educational history, past mental health, substance use, living status, history of domestic violence, participation in tribal activities, knowing anyone who died by suicide in their lifetime, and number of indexed events	RF, SVM, Lasso-R, RLR	Repeated cross-validation with 10 iterations (I)	Classification	Not reported	AUC: 0.87
Huber (2020) [38]	Migrant status	653 variables	LR, DTs, SVM, and naive Bayes	5-fold cross-validation (I)	Classification	Not reported	DT Accuracy: 74.5%; AUC: 0.75
Khatua (2021) [40]	Tweets that fall into 3 themes: generic views, initial struggles, and subsequent settlement	Tweets	Bi-LSTM, CNN, BERT	Training and testing	Classification	Python	F1-Score: 61.61–75.89%
Liu (2021) [43]	MH diagnosis from EHR	Copy number variation	Multi-layer perceptron	Two-fold random shuffle test validation (I)	Classification	Python; Scikit-learn package	Accuracy: 65.7%
Liu (2021) [42]	ADHD diagnosis	Copy number variation	Multi-layer perceptron	Two-fold random shuffle test validation (E)	Classification	Python; Scikit-learn package	Accuracy: 75.4%

Abbreviations: Logistic regression (LR), Ridge logistic regression (RLR), Least Absolute Shrinkage and Selection Operator, (Lasso-LR), random forests (RF), deep learning neural networks (DNNs), Ensemble learning (EL), Lasso-Regression (Lasso-R), gradient boosting machines (GBM), Natural language processing (NLP), Artificial Neural Network (ANN), decision trees (DTs), support vector machines (SVM), Bidirectional Long Short-Term Memory (Bi-LSTM) and Convolutional neural network (CNN), Bidirectional Encoder Representations from Transformers (BERT), area under the receiver operating characteristic Curve (AUC), Root-mean-square error (RMSE)

The publications used a range of ML models, from one [31–35, 42, 43] to 16 [36]. In studies where multiple ML models were used, the aim was often to compare the models to determine the best predictive power. For example, Acion et al. compared 16 models and evaluated them using the area under the receiver operating characteristic curve (AUC) to classify substance use disorder treatment success in Hispanic patients [36]. Huber et al. compared five different ML algorithms, including decision trees, support vector machines, naïve Bayes, logistic regression, and K-nearest neighbor, to determine the model with the best predictive power for classifying schizophrenia spectrum disorders in migrants [38]. Two of the studies used linear regression [33, 34]. All of the studies developed custom models to meet their study aims. The most common programs used in these studies were R [31, 36], SPSS [32, 34], and Python [40, 42, 43].

Predictors that were included in the modeling were sociodemographic characteristics [32, 34, 36–39], and some also included MH variables and experiences [31, 32, 34, 36–39] collected from EHRs or surveys. One study first determined which of the included 653 input variables (including sociodemographic data, childhood/adolescence experiences, psychiatric history, past criminal history, social and sexual functioning, hospitalization details, prison data, and psychopathological symptoms) were the best predictor variables and trained a final ML algorithm using only those [38].

Two studies did not report the best algorithm performance [37, 41]. For the other studies, accuracy and AUC were commonly reported. For example, Acion et al. classified substance use disorder treatment success in Hispanic patients and found that the AUC of studied models ranged from 0.793 to 0.820, with the ensemble method achieving an AUC of 0.820, which was not significantly better than the traditional logistic regression model’s AUC of 0.805 [36]. Huber et al. identified a tree algorithm that differentiated native Europeans and non-European migrants with schizophrenia with an accuracy of 74.5% and a predictive power of AUC = 0.75 [38]. In Liu et al., the trained ML model had an accuracy of 78% in predicting ADHD in African American patients [42]. In a similar study to classify ADHD, depression, anxiety, autism, intellectual disabilities, speech/language disorder, developmental delays, and oppositional defiant disorder in African Americans, the model had an accuracy of 65% in distinguishing patients with at least one MH diagnosis from controls [43]. A second prediction model aimed at predicting the diagnosis of two or more MH disorders had a low accuracy level, with an exact match rate of 7.2–9.3% [43]. Khatua and Nejdl [40] analyzed tweets acquired from Twitter feeds from self-identified refugees and categorized them into themes of the immigrant struggle with an accuracy of 61.61% and 75.89%.

The included studies also used p-values to assess their ML algorithms. Goldstein and Bailey utilized multivariable logistical regression to examine the relationship between experienced discrimination and suicidal ideation in Hispanic patients [37]. They found that 19.0% of Hispanic patients who experienced discrimination also experienced suicidal ideation, compared to 11.5% of patients who did not experience discrimination (*p* = 0.001). Moreover, Hispanic patients had 1.72 greater odds of having suicidal thoughts if they experienced discrimination compared to those who did not (*p* = 0.003). A study by Erol and Seçinti used regression analysis to study the relationship between PTSD and depression and various predictors in adolescent refugee minors [34]. They found that moderate and severe changes in family income level and stress in food access predicted depression scores and PTSD symptoms (*p* < 0.01). Drydakis [33] used random effects models to estimate the relationship between the number of mobile applications that facilitate immigrants’ societal integration and immigrants’ integration, health, and mental health [28]. The results showed a negative association between the number of standard m-Integration applications and adverse MH status (*p* < 0.01). Accuracy was also measured using importance and normalized importance [32], Root-mean-square error (RMSE) [31], and Least Absolute Shrinkage and Selection Operator (LASSO) coefficients [35].

### Cross validation

Six studies used internal cross-validation methods [31, 35, 36, 38, 39, 43]. Only one study used an external data set to validate their ML algorithm [42]. That external validation of the algorithm reduced the accuracy of their algorithm from 78% to 70–75% [42]. Almost half of the included publications did not use or discuss their cross-validation method [32–34, 37, 41].

### Gaps in ML for MH in vulnerable populations

Our analysis reveals significant gaps in the use of machine learning to address mental health in vulnerable populations such as immigrants, refugees, migrants, and racial and ethnic minorities. Key issues include the underrepresentation of these groups in training datasets, leading to biased algorithms, and the lack of adapted models. Additionally, integration challenges within healthcare systems that serve these populations, combined, significantly hinder the effectiveness and ethical application of ML technologies. Addressing these gaps is crucial for ML to improve MH outcomes equitably.

## Discussion

This exploratory scoping review explores the application of ML in MH research, focusing on vulnerable populations including immigrants, refugees, and ethnic minorities. Our findings reveal that ML is increasingly used to enhance MH diagnostics, screening, and interventions.

In recent years, there has been significant interest in the potential of ML to transform the field of MH research [29]. Studies examining ML models in a variety of clinical settings indicate that ML may outperform traditional statistical models, especially as they relate to prognosis or predicting behavior [44–48].

While ML algorithms can effectively handle large volumes of EHR data for risk prediction, it’s important to note that they still require significant manual input and optimization [47, 49]. Unlike traditional statistical techniques that often involve manual selection and imputation of specific variables, ML models can potentially consider a broader range of data points [44, 48]. However, these models typically require extensive tuning, which involves considerable manual labor and decision-making on the part of developers. Additionally, ML can sometimes capture more intricate, non-linear relationships without the need for explicit specification of interaction terms.

It’s important to note that ML encompasses a broad range of techniques, including simple linear regression, which is also used in traditional statistical analysis. The advantage of more advanced ML models often lies in their ability to automatically detect and utilize complex interactions and non-linear relationships in high-dimensional data, potentially leading to improved predictive performance in certain scenarios, including the need for careful model selection, hyperparameter tuning, and validation to ensure reliable and generalizable results [50].

Recent advances in computational power and software availability have enabled researchers to reach new audiences and demonstrate the clinical value of ML. In particular, some studies have aimed to inform clinicians about the methods and applications of ML in the context of psychotherapy [51]. However, while many of the reviewed papers provide proof-of-concept for the potential use of ML algorithms to address MH concerns, our review finds that the clinical application of these models for classifying and predicting MH disorders is still under development.

Despite ML’s great interest and potential to transform MH research, few researchers have focused on specific and marginalized populations. In reviewing hundreds of articles on MH and ML, we found only a handful of studies specifically targeting immigrants, migrants, refugees, and/or racial and ethnic minorities. Many researchers simply included race as a variable in their models rather than designing ML algorithms to analyze these specific groups of individuals [52, 53]. Moreover, as noted by Maslej et al. [30], most studies that considered African American and White samples used self-reported race or ethnicity or did not describe how this information was collected and thus were excluded from our analysis.

There is still a wide gap in health disparities that persist in accessing quality MH services and outcomes. These gaps primarily concern the limited diversity of populations, the lack of research on complex MH outcomes, and the challenges associated with integrating ML tools in healthcare settings. The current lack of ML models tailored to specific populations presents opportunities and challenges. On the one hand, it can help prevent the perpetuation of health disparities that arise when models built on majority populations are used to misclassify minorities [54]. Performance differences in ML exist for different populations, especially with genomic data. For instance, one study externally validated their algorithm [42] on White Americans rather than African Americans and found that their algorithm’s accuracy decreased. On the other hand, this lack of tailored models highlights the opportunity for researchers and clinicians to bridge the gap between what is known about majority populations and what is yet to be uncovered in other populations. Training ML models on other groups could expedite this process without being too resource-intensive. By proposing future research directions aimed at closing these gaps, we highlight the need for more inclusive data collection, enhanced algorithm training that reflects diverse patient experiences, and comprehensive evaluations of ML tools in real-world clinical settings.

One of the most common challenges in utilizing ML techniques to build classifiers for MH is the use of small sample sizes, which may limit the representation of the entire population and impact the generalizability of the classifier’s accuracy estimate. This can be a practical limitation due to resource constraints in real-world clinical or diagnostic settings. However, researchers need to understand that using ML alone cannot address this issue [26]. Most ML methods rely on supervised learning models, which are successful due to the abundance of training data. However, this training data requires human annotation, which can be time-consuming and costly. In the case of MH, there are insufficient publicly annotated datasets, making the quality of the data a significant concern for developing reliable models [53].

Another challenge of using ML for behavioral diagnosis is validating the classification algorithms against questionnaires or clinical diagnoses, which are known to have self-report biases and are not completely accurate. This highlights the lack of established best standards in the diagnosis process for mental disorders and other psychiatric conditions [55]. Future directions include the development of more robust and generalizable algorithms that can improve prediction capabilities. ML can be leveraged to understand the prevalence of MH conditions at a population level by using open-source and freely available data, which can be more accurate and less labor-intensive than traditional surveys. Furthermore, ML can enable the study of MH in children and adolescents in innovative ways [35, 43]. The application of these models can be expanded to different sources and sample sizes, potentially leading to a rapid increase in their use in clinical settings.

The growing application of ML in mental health research presents several key implications. First, there’s a critical need for more focused research on vulnerable populations, including immigrants, refugees, and racial/ethnic minorities, to address potential biases and unique challenges [56]. Second, while promising, the clinical implementation of ML for MH diagnostics and prediction is still in its early stages, necessitating further validation and strategies to overcome integration barriers [28]. Lastly, the lack of appropriate cross-validation techniques in many studies highlights the urgent need for more rigorous methodological approaches to ensure the reliability and real-world applicability of ML models in mental health contexts [57]. Addressing these implications is crucial for realizing the full potential of ML in advancing mental health research and practice.

ML exhibits varying degrees of effectiveness across MH conditions, largely influenced by the availability of data and the complexity of symptoms. However, results have been mixed. Nemesure et al. [58] used ML to predict depression and anxiety, achieving moderate success but below clinical standards for diagnostics. These findings show both the potential and current limitations of ML in mental health. While ML can process large datasets and potentially uncover subtle patterns, achieving clinically acceptable accuracy remains challenging. Further research is needed to improve ML models before they can be widely applied in mental health diagnosis and treatment planning. Conversely, in complex disorders such as schizophrenia and bipolar disorder, while ML can predict episodes, the variability in symptoms poses challenges for model accuracy [59]. Neurodegenerative diseases, such as Alzheimer’s, also benefit from ML in early detection, though the gradual progression of symptoms limits its immediate utility [60]. In substance use disorders, ML’s ability to track behavioral patterns offers the potential for predicting relapse [61]. Future research should thus focus on enhancing data collection and refining ML models to accommodate the specific needs of each condition while addressing ethical concerns. Also, there is a critical need for addressing algorithmic bias within healthcare to prevent disparities among racial and ethnic minority groups [49]. Chin et al. underline a framework for mitigating bias across an algorithm’s lifecycle, from problem formulation to deployment and monitoring, underscoring the importance of transparency, accountability, and community engagement in ML development [49].

There is also potential for the future application of ML and natural language processing (NLP) approaches to infer psychological well-being and detect CMDs in marginalized individuals based on social media posts on platforms like Facebook and Twitter. Researchers must implement diagnostic criteria and tools that are precise and suitable for various online populations. Personal information, such as sociodemographic characteristics and behavioral aspects, must be collected by ethical considerations. These inferences can create online platforms that provide health information, support, and tailored interventions. Currently, the computational techniques and evaluations employed for collecting, processing, and utilizing online written data remain scattered throughout academic literature [62]. Moreover, this potential is limited by factors such as class imbalance, noisy labels, and text samples that are either too long or too short, which can lead to performance and stability issues. The diversity of writing styles and semantic heterogeneity in different data sources can also cause a lack of robustness in model performance. Standardizing these measures can allow for the development of scalable approaches for automated monitoring of public psychological health in the future [43].

This review had limitations, including the possibility of missing relevant studies due to specificity in search terms. Future studies should consider using broader search terms to address these limitations. Additionally, the ethical and social implications of using ML in MH, including the potential for perpetuating existing biases and social determinants of health, should be carefully considered. Discussing ethical concerns is important when utilizing textual data related to MH, given the significance of privacy and security of personal information, particularly health data.

## Conclusions

In conclusion, ML can potentially transform how we understand mental health, particularly among vulnerable populations. Immigrants and refugees face unique challenges related to migration and resettlement that can negatively impact their MH status, including poverty, discrimination, and exposure to trauma. African Americans and Hispanics in the US also have higher persistence and disability from mental illness. This review has found that, to date, few studies have used ML to predict and classify MH in these populations, despite the wide gap in health disparities that persist in accessing quality MH services and outcomes. The use of big data and ML algorithms in the health sciences is increasing and holds promise, but more study of ML applications in MH is warranted.

## Electronic supplementary material

Below is the link to the electronic supplementary material.

Supplementary Material 1

Supplementary Material 2

## Data Availability

The search strings and data sets used and/or analyzed during the current study are available from the corresponding author upon reasonable request. A preprint of this manuscript may also be found online.
