# Supplementary material for: Machine learning applications in studying mental health among immigrants and racial and ethnic minorities: an exploratory scoping review
Source: BMC Med Inform Decis Mak. 2024 Oct 10;24:298. doi: 10.1186/s12911-024-02663-4 (PMC11468366; doi:10.1186/s12911-024-02663-4)
Supplement: Supplementary file 2 — Supplementary Material 2 [file 12911_2024_2663_MOESM2_ESM.docx]

**Appendix A.** Database queries and keywords

EMBASE (Emtree and keywords):

('migrant'/exp/mj OR migrant*:ti,ab OR refuge*:ti,ab OR immigra*:ti,ab OR emigra*:ti,ab OR asylum:ti,ab OR minorit*:ti,ab OR latin*:ti,ab OR hispanic:ti,ab OR asia*:ti,ab OR native:ti,ab OR 'american indian'/dm OR black:ti,ab OR 'pacific islander':ti,ab OR  indigenous':ti,ab OR ' aboriginal':ti,ab OR ' First Nations':ti,ab)

AND

('mental health'/exp/mj OR (mental:ti,ab AND health:ti,ab) OR 'anxiety disorder'/dm OR 'attention deficit hyperactivity disorder'/dm OR 'bipolar disorder'/dm OR 'cognitive defect'/dm OR 'dementia'/dm OR 'depression'/dm OR 'fatigue'/dm OR 'generalized anxiety disorder'/dm OR 'major depression'/dm OR 'memory disorder'/dm OR 'mental disease'/dm OR 'mild cognitive impairment'/dm OR 'mood disorder'/dm OR 'obsessive compulsive disorder'/dm OR 'posttraumatic stress disorder'/dm OR 'psychosis'/dm OR 'schizophrenia'/dm OR 'sleep disorder'/dm OR 'suicide'/dm)

AND

('artificial intelligence'/exp/mj OR 'machine learning'/exp/mj OR 'deep learning'/exp/mj OR 'algorithm') AND [english]/lim AND [abstracts]/lim AND [article]/lim OR [data papers]/lim)

PubMed (MeSH and keywords):

("refugees"[MeSH Terms] OR "refuge*"[Title/Abstract] OR "migrant*"[Title/Abstract] OR "immigra*"[Title/Abstract] OR "emigra*"[Title/Abstract] OR "asylum"[Title/Abstract] OR "minorit*"[Title/Abstract] OR "latin*"[Title/Abstract] OR "hispanic"[Title/Abstract] OR "asia*"[Title/Abstract] OR "native"[Title/Abstract] OR "american indian"[Title/Abstract] OR "black"[Title/Abstract] OR "pacific islander"[Title/Abstract] OR “indigenous” [Title/Abstract] OR "aboriginal" [Title/Abstract] OR "First Nations" [Title/Abstract])

AND

("mental health"[MeSH Terms] OR "mental health"[Title/Abstract]) OR (“anxiety disorder” [Title/Abstract] OR “attention deficit hyperactivity disorder” [Title/Abstract] OR “bipolar disorder” [Title/Abstract] OR “cognitive defect” [Title/Abstract] “dementia” [Title/Abstract] OR “depression” [Title/Abstract] OR “generalized anxiety disorder” [Title/Abstract] OR “major depression” [Title/Abstract] OR “memory disorder” [Title/Abstract] OR “mental disease” [Title/Abstract] OR “mild cognitive impairment” [Title/Abstract] OR “mood disorder” [Title/Abstract] OR “obsessive compulsive disorder” [Title/Abstract] OR “posttraumatic stress disorder” [Title/Abstract] OR “psychosis” [Title/Abstract] OR “schizophrenia” [Title/Abstract] OR “sleep disorder” [Title/Abstract] OR “suicide” [Title/Abstract])

AND

((((Machine Learning[MeSH Terms]) OR Machine Learning[Title/Abstract]) OR Artificial Intelligence[MeSH Terms]) OR Artificial Intelligence[Title/Abstract] OR deep learning[Title/Abstract] OR algorithm[Title/Abstract])

PsycINFO (MeSH and keywords):

((("MeSH:refugees") OR (abstract:"refuge*") OR (abstract:"migrant*") OR (abstract:"immigra*") OR (abstract:"emigra*") OR (abstract:"asylum") OR (abstract:"minorit*") OR (abstract:"latin*") OR (abstract:"hispanic") OR (abstract:"asia*") OR (abstract:"native") OR (abstract:"american indian") OR (abstract:"black") OR (abstract:"pacific islander") OR (abstract:" indigenous") OR (abstract:" aboriginal") OR (abstract:" First Nations")))

AND

(("MeSH:mental health") OR (abstract:"mental health") OR (abstract:"anxiety disorder") OR (abstract:"attention deficit hyperactivity disorder") OR (abstract:"bipolar disorder") OR (abstract:"cognitive defect") OR (abstract:"dementia") OR (abstract:"depression") OR (abstract:"generalized anxiety disorder") OR (abstract:"major depression") OR (abstract:"memory disorder") OR (abstract:"mental disease") OR (abstract:"mild cognitive impairment") OR (abstract:"mood disorder") OR (abstract:"obsessive compulsive disorder") OR (abstract:"posttraumatic stress disorder") OR (abstract:"psychosis") OR (abstract:"schizophrenia") OR (abstract:"sleep disorder") OR (abstract:"suicide"))

AND

((("MeSH:Machine Learning") OR (abstract:"Machine Learning") OR (abstract:"Artificial Intelligence") OR (abstract:"deep learning") OR (abstract:"algorithm")))

Google Scholar:

("refugees" OR "refuge*" OR "migrant*" OR "immigra*" OR "emigra*" OR "asylum" OR "minorit*" OR "latin*" OR "hispanic" OR "asia*" OR "native" OR "american indian" OR "black" OR "pacific islander" OR “indigenous” OR “aboriginal” OR “First Nations”)

AND

("mental health" OR "anxiety disorder" OR "attention deficit hyperactivity disorder" OR "bipolar disorder" OR "cognitive defect" OR "dementia" OR "depression" OR "generalized anxiety disorder" OR "major depression" OR "memory disorder" OR "mental disease" OR "mild cognitive impairment" OR "mood disorder" OR "obsessive compulsive disorder" OR "posttraumatic stress disorder" OR "psychosis" OR "schizophrenia" OR "sleep disorder" OR "suicide")

AND

("Machine Learning" OR "Artificial Intelligence" OR "deep learning" OR "algorithm")
